# The effects of cannabidiol and its main metabolites on human neural stem cells

**DOI:** 10.3389/ebm.2025.10608

**Published:** 2025-06-13

**Authors:** Leah E. Latham, Qiang Gu, Shuliang Liu, Cheng Wang, Fang Liu

**Affiliations:** Division of Neurotoxicology, National Center for Toxicological Research/Food and Drug Administration, Jefferson, AR, United States

**Keywords:** CBD, 7-OH-CBD, 7-COOH-CBD, THC, neural stem cells

## Abstract

Cannabidiol (CBD) has been used for different purposes by different populations in recent years. When consumed by pregnant women, CBD can pass through the placenta and enter the fetal blood stream. There is concern over adverse effects of fetal exposure to CBD and its major metabolites (7-OH-CBD and 7-COOH-CBD). In the present study, human neural stem cells (NSCs) were treated with CBD and its metabolites at different concentrations for various durations to understand how the drug may affect fetal brain development. NSCs were also treated with delta-9 tetrahydrocannabinol (THC) for comparison purposes. CBD, 7-OH-CBD and 7-COOH-CBD dose-dependently reduced NSC viability. CBD and 7-OH-CBD reduced NSC number at the G1 phase. A 24 h exposure did not cause significant change in NSC proliferation. At concentrations comparable to those detected in human blood, longer exposures to CBD, 7-OH-CBD and 7-COOH-CBD caused more obvious cell death. After NSCs differentiation, CBD treatment reduced GFAP and cannabinoid receptor 2 (CB2) expression. THC treatment reduced the GFAP expression, but the change in CB2 expression did not reach statistical significance. The expression of cannabinoid receptor 1 (CB1) and beta-tubulin III were not significantly altered by drug exposures. The study demonstrated that clinically relevant concentrations of CBD, 7-OH-CBD and 7-COOH-CBD affect basic physiological features of human NSCs. After NSC differentiation, the reduced expression of CB2 receptors and GFAP on differentiated cells further indicated the vulnerability of developing central nervous system to CBD and THC. These data will help to contextualize *in vivo* neurodevelopmental studies that may not accurately model human metabolite profiles of CBD.

## Impact statement

There is a need to understand the effect of CBD on the developing brain. The current work demonstrates that exposure to CBD during early development poses a risk to the human developing brain. The work provides direct evidence on the adverse effects of 7-OH-CBD and 7-COOH-CBD on the human developing brain, helping to differentiate the effects of CBD from those of its major metabolites of 7-OH-CBD and 7-COOH-CBD. CBD and its major metabolites additively affect the developing central nervous system. The current study observes the effects of CBD and its metabolites on the brain cells, providing evidence that helps to distinguish the effects of CBD from those of its metabolites *in vivo*.

## Introduction

Cannabidiol (CBD) is a non-intoxicating compound found in the plant Cannabis Sativa. Like with delta-9 tetrahydrocannabinol (THC), it naturally occurs in Cannabis Sativa. In 2018, the Farm Bill removed hemp which contains “no more than 0.3% THC on a dry weight basis” from marijuana (containing high levels of THC to have psychoactive effects, also called “cannabis”) in the Controlled Substances Act. Hemp products, especially those that contain CBD have rapidly proliferated. Preclinical and clinical studies indicate that CBD may have some therapeutic properties such as antidepressant-like, anxiolytic-like, anti-inflammatory, and antioxidative effects [1–5]. A recent clinical trial reported that CBD reduced cue-induced craving and anxiety of patients with opioid addition [6], highlighting the potential of CBD-based therapies for treating opioid use disorder. Currently, only one CBD product (Epidiolex^®^) is approved by the U.S. Food and Drug Administration (FDA) for treating refractory epilepsy in children. Despite being widely available, no other CBD products are approved for the treatment of medical conditions. A survey that included a random sample of 2,543 adults from all 50 U.S. states and the DC area showed 20% of 18-29-year-old adults and 16% of 30–49-year-old adults used a CBD product in 2019 [7]. Another survey with data collected from 2,000 Americans showed 33% of American adults used CBD in 2020 [8]. Most people use CBD to medicate themselves simply due to the perception that it is natural and safer than other drugs. However, there is no data demonstrating the CBD products are safe and efficacious for the treatment of medical conditions other than seizure in very select populations, and CBD is not risk free. CBD may be hepatotoxic [9–11], actively interact with other drugs [12, 13], suppress immune function [14, 15], and adversely affect the male reproductive system [16, 17].

In humans, CBD is rapidly metabolized [18]. Among the numerous CBD metabolites, 7-COOH-CBD is the most abundant in plasma, even more so than the parent compound. In humans, the second most abundant metabolite is 7-OH-CBD, whose concentration is comparable to CBD in plasma. While 7-OH-CBD has been reported to be bioactive [18, 19], whether 7-COOH-CBD has any bioactivity is not yet fully determined.

In the general population people use CBD for a variety of reasons. Many pregnant people report self-medicating with CBD to treat nausea, anxiety, and pain. When consumed by pregnant people, CBD can pass through the placenta [20], enter fetal blood circulation and directly interact with fetal organs. Moreover, CBD may enhance placenta permeability to other chemicals and increase the exposure of fetuses to those compounds [21]. Detection of CBD metabolites in meconium suggests that CBD is metabolized by fetuses, or that the metabolites can cross the placenta [22].

The endocannabinoid system is widely expressed in the central nervous system (CNS). It has an essential role in brain development and regulates and controls synaptic activity by releasing endogenous cannabinoids to interact with related receptors [23, 24]. CBD affects both developing and mature brains via various mechanisms, serving as a modulator of the endocannabinoid system [25]. CBD consumption during pregnancy causes fetal exposure to CBD which can accumulate in the brain due to its lipophilicity [26]. Adverse effects of CBD on the developing animal brain have been reported recently [27, 28]. With the high concentrations of 7-OH-CBD and 7-COOH-CBD in plasma, it merits further investigation to understand if the two most abundant metabolites have any effects on the human developing brain, which may contribute to the effects of CBD. Moreover, the decriminalization and legalization of cannabis for both medical and recreational use in many states in the US has caused a spike in THC consumption. THC is the most widely used illegal drug by pregnant women. It has been demonstrated that prenatal THC exposure adversely affects neurodevelopment [29], causing hyperactivity, cognition impairment, etc. in childhood [20, 30]. In the present study, we exposed human neural stem cells (NSCs) and cells that were differentiated from NSCs to CBD, 7-OH-CBD, 7-COOH-CBD and THC to assess their effects on NSC proliferation, viability and cell cycles, and the gene expression of some representative molecules on differentiated cells to get a basic idea on how they may affect brain biology at an early developmental stage.

## Materials and methods

### Test chemicals

CBD, 7-COOH-CBD (7-carboxy-CBD), 7-OH-CBD (7-hydroxy-CBD) and THC were purchased from Purisys (Athens, GA). CBD and the metabolites were pure; and the purity of THC was more than 95%, as stated by the manufacturer. They were dissolved in Dimethyl sulfoxide (DMSO, MilliporeSigma, St. Louis, MO) and stored in a −20°C freezer.

### Human neural stem cell (NSC) culture

Human NSCs purchased from PhoenixSongs Biologicals (Branford, CT) were used in the study. These de-identified cells were derived from the hippocampus of human fetal brain. Media for NSC proliferation (named “growth medium”) and differentiation (named “differentiation medium”) were purchased from the same vendor. These cells have been confirmed to be NSCs and capable of differentiating into neurons, astrocytes and oligodendrocytes in our previous studies [31, 32]. The cells were seeded on laminin-coated dishes of 10 cm in diameter at a density of 4.5 × 10^4^/cm^2^ and cultured with growth medium to promote NSC proliferation in a humidified incubator at 37°C with 5% CO_2_. The same cell density of 4.5 × 10^4^/cm^2^ was applied when NSCs were seeded on 96-well plates for assays. More than 95% of the seeded cells were viable 24 h after seeding. Oxygen level in the incubator was controlled at 4% as the vendor recommended to promote NSCs to differentiate into neurons. To induce NSC differentiation, NSCs were cultured in differentiation medium. After 3 days differentiation, these cells were treated with CBD, 7-OH-CBD, 7-COOH-CBD and THC in differentiation medium for 6 days before harvested. NSCs from passage 12 to 15 were used for experiments.

### LDH release assay

Lactate dehydrogenase (LDH) release assay (Roche Applied Science, Indianapolis, IN) was performed as previously reported [33, 34] to determine cytotoxicity after chemical exposures for 1, 3, 5, and 7 days.

### 5-ethynyl-2′-deoxyruidine (EdU) incorporation assay

NSC proliferation rate was measured using an EdU staining kit [Click-iT^®^ EdU Alexa Fluor^®^ High-throughput Imaging (HCS) Assay, Invitrogen, Carlsbad, CA] after 24-h exposure to the chemicals, as the manufacturer instructed.

### Flow cytometric analysis of cell cycle

Cell cycle status was analyzed using flow cytometry, by quantifying DNA content with DNA-binding dye propidium iodide (PI, MilliporeSigma). After 24 h exposure to drugs, human NSCs were harvested, fixed and permeabilized in cold 70% ethanol. To ensure PI would stain DNA only, cellular RNA was digested with RNase A at 37°C for 1 h before DNA staining with PI. A LSRFortessa™ flow cytometer with FACSDiva™ software (BD Biosciences, San Jose, CA) was used to acquire PI signals and FCS Express (*De Novo* software, Pasadena, CA) was used to distinguish cells in each cell cycle phase. A total of 50,000 events were recorded on the flow cytometer.

### Glutathione (GSH) assay

After 7 days drug exposures, the oxidative status of NSCs was assessed using GSH-Glo™ Glutathione assay (Promega, Madison, WI) as the manufacturer described. Briefly, NSCs cultured in 96-well plates were incubated with 1X GSH-Glo™ Reagent at room temperature, followed by incubation with Luciferin Detection Reagent and luminescence measurement.

### Annexin V labeling for flow cytometry

To understand whether CBD, its metabolites or THC induced apoptosis or necrosis, human NSCs were labeled with Annexin V and PI (BD Biosciences) as manufacturer instructed after 24 h drug exposure. In brief, collected NSCs were washed with cold PBS, resuspended in Binding Buffer, and incubated with FITC Annexin V and PI, followed by flow cytometry analysis.

### Terminal deoxynucleotidyl transferase dUTP nick-end labeling (TUNEL) assay

After 24 h exposure to CBD, its metabolites and THC, human NSCs were fixed with paraformaldehyde for TUNEL assay, using TUNEL Andy Fluor™ 488 Apoptosis Detection Kit (ABP Biosciences, Rockville, MD) as previously described [35].

### Western-blots of β-tubulin III, glial fibrillary acidic protein (GFAP), oligodendrocyte myelin glycoprotein (OMG), capase 3 and cannabinoid receptors 1 and 2 (CB1 and CB2)

Western-blots of β-tubulin III, GFAP, OMG, caspase 3 (pro-caspase 3 and active caspase 3), CB1 and CB2 were conducted using Jess™ (ProteinSimple Inc.), whose protein separation principal is based on capillary electrophoresis technology. Protein analysis was performed following the protocol provided by ProteinSimple Inc. In brief, protein samples (0.5 mg/ml) were mixed with a sample buffer containing 200 mM dithiothreitol (DTT) and fluorescent standards (4:1 vol/vol), and denatured at 95°C for 5 min. The protein samples were loaded into capillaries, separated, immobilized, incubated with respective primary antibodies (1:50, β-tubulin III, GFAP and CB1: MilliporeSigma; OMG and CB2: Abcam; caspase 3: Novus Biologicals) for 1 h, washed, and then incubated with horse radish peroxidase-conjugated anti-rabbit (GFAP, CB1 receptor, CB2 receptor and caspase 3) or anti-mouse (β-tubulin III) secondary antibodies for 1 h. After washing, the capillaries were incubated with the luminol-S/peroxide substrates, and chemiluminescence signals were captured using a charge-coupled device (CCD) camera. After the chemiluminescence signals of the target protein in each capillary were captured, the chemiluminescence signals were stripped using a RePlex kit (ProteinSimple Inc.). Then, total proteins in each capillary were determined using the Simple-Western Total Protein Detection Module (ProteinSimple Inc.), which is a chemiluminescence based total protein assay kit. The chemiluminescence signals of the target protein and the total protein in each capillary were measured using the Compass software (ProteinSimple Inc.). The signal intensity of the target protein in each capillary were normalized automatically by the Compass software based on the signals of the total proteins in that capillary. The normalized signal intensity of the target protein represents the relative abundance of the target protein. ANOVA test was used to compare the relative abundance of each target protein among the different treatment groups.

### Statistical analysis

Data were analyzed with GraphPad Prism 9 (GraphPad Software Inc., San Diego, CA) using one-way ANOVA followed by Dunnett’s *post hoc* test, and expressed as mean ± SD. Each experiment was repeated at least three times independently. It was statistically different when a *p* value is less than 0.05.

## Results

### Cytotoxic effects of drugs

**FIGURE 1:**
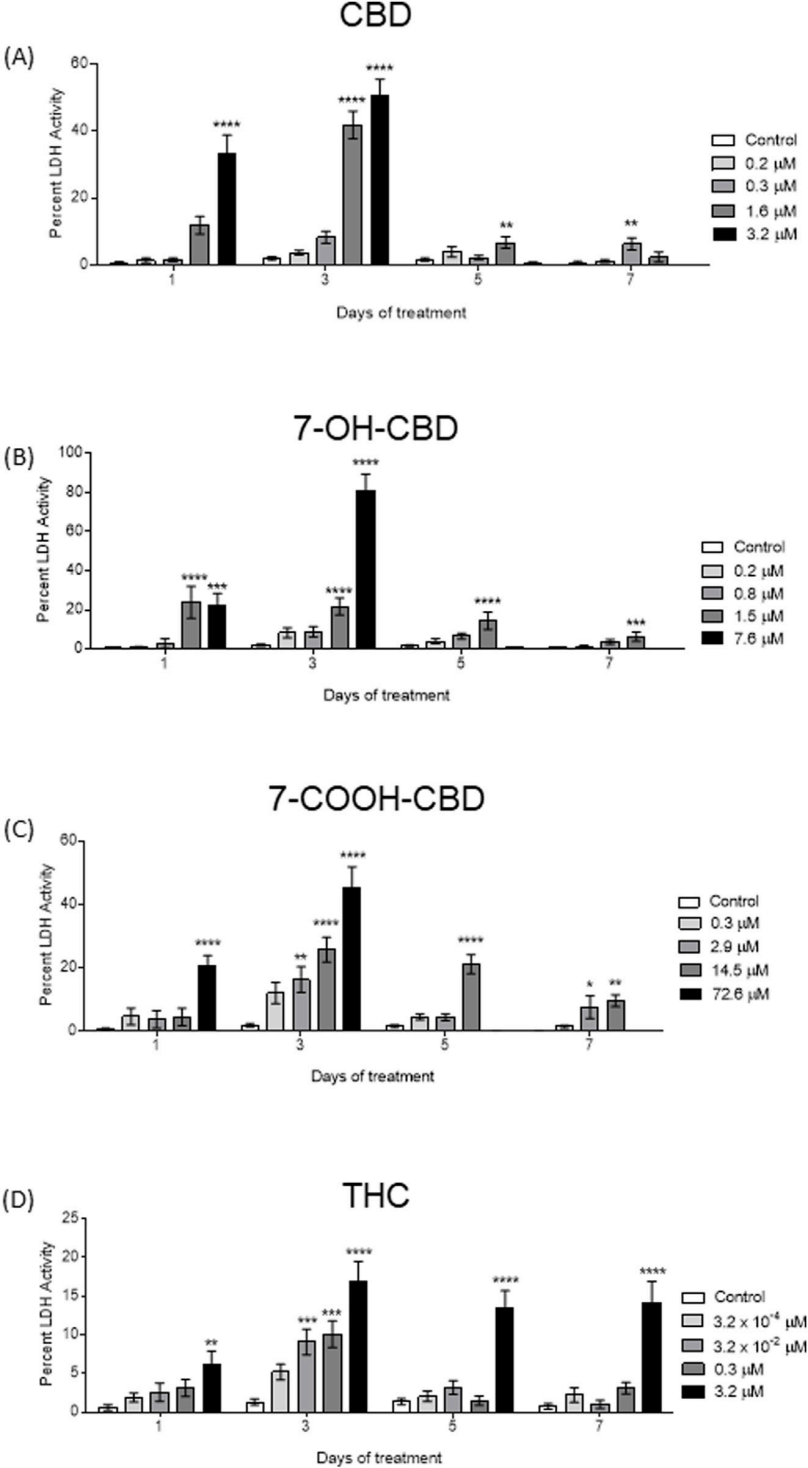
LDH release assay after CBD, 7-OH-CBD, 7-COOH-CBD and THC exposures for 1, 3, 5 and 7 days. **(A)** Exposure to 3.2 µM CBD significantly increased LDH release after 1 day and 3 days exposures; no obvious LDH release was detected after 5 days and 7 days exposures. The cytotoxic effects of 1.6 µM CBD were not significant until after 3 days exposure. Exposure to 0.3 µM CBD for 7 days caused an increase of LDH release. **(B)** 7-OH-CBD at 1.5 µM caused LDH release after 24 h exposure and continued through the entire experiment. No cells survived in 7.6 µM 7-OH-CBD group after 5 days exposure. **(C)** 7-COOH-CBD of 72.6 µM increased LDH release after 1 day and 3 days exposures; no obvious LDH release was detected after 5 days and 7 days exposures due to loss of cell. At 14.5 µM, 7-COOH-CBD induced higher LDH release after 3, 5 and 7 days, while 2.9 µM 7-COOH-CBD only show significant effects after 7 days exposure. **(D)** Exposure to 3.2 µM THC increased LDH release starting from the first day of exposure. At lower concentrations, 3.2 × 10^-2^ μM THC and 0.3 µM THC caused a transient LDH increase after 3 days exposure. *P < 0.05; **P < 0.01; ***P < 0.001; ****P < 0.0001, n = 4–6. The experiment was repeated at least three times independently.

Human NSCs were exposed to a wide range of concentrations of CBD, 7-COOH-CBD, 7-OH-CBD and THC, which were selected according to the concentrations detected in human blood [18]. The LDH release assay revealed that CBD, its metabolites, and THC increased LDH release in a dose- and duration-dependent manner (Figure 1), suggesting these drugs caused cell death.

After 24 h exposure to 3.2 µM CBD, LDH release was significantly increased by 32.7% compared with control; the increase reached to 48.9% after 3 days exposure. No obvious LDH release was observed on day 5 and 7 due to the loss of cells in this group. CBD at 1.6 µM caused an increase of LHD release after 24 h exposure, but it did not reach statistical significancy until after 3 days of exposure, when the LDH release was significantly elevated to 41.8%, and another significant increased LDH release after 5 days exposure. It was noticed that the surviving cells in the 1.6 µM CBD-treated group were not enough to make a significant change of LDH release after 7 days exposure. In addition, exposure to 0.3 µM CBD resulted in a significant increase of LDH release by 5.7% after 7 days exposure (Figure 1A).

The toxic effect of 7.6 µM 7-OH-CBD was revealed by the elevated LDH release of 21% after 24 h exposure (Figure 1B). It induced an 79.3% increase of LDH release after 3 days exposure. There was no viable cell left in the 7.6 µM 7-OH-CBD -treated group afterwards, and no obvious LDH release was detected. 7-OH-CBD at 1.5 µM caused LDH release through the entire exposure time course: the elevated release reached 23.8%, 21.8%, 14.4%, and 6.3% when measured after 1, 3, 5, and 7 days exposure. Lower concentrations of 7-OH-CBD (0.8 µM and 0.2 µM) did not induce a significant increase of LDH release (Figure 1B).

Being the most abundant CBD metabolite, 7-COOH-CBD at 72.6 µM increased LDH release by 20.3% after 24 h exposure and increased by 43.7% after 3 days exposure. Lower concentrations of 7-COOH-CBD also stimulated LDH release after longer exposure: a continuous elevation of LDH release was observed in 14.5 µM 7-COOH-CBD-treated group after 3 days exposure, reaching 23.9%, 1.5%, and 9.7% after 3, 5, and 7 days exposure (Figure 1C). Higher levels of LDH release were detected in the 3.2 µM THC group throughout the exposure period. An elevation of 5.7%, 15.7%, 12.3% and 13.5% occurred after 1, 3, 5 and 7 days exposure, respectively (Figure 1D).

**FIGURE 2:**
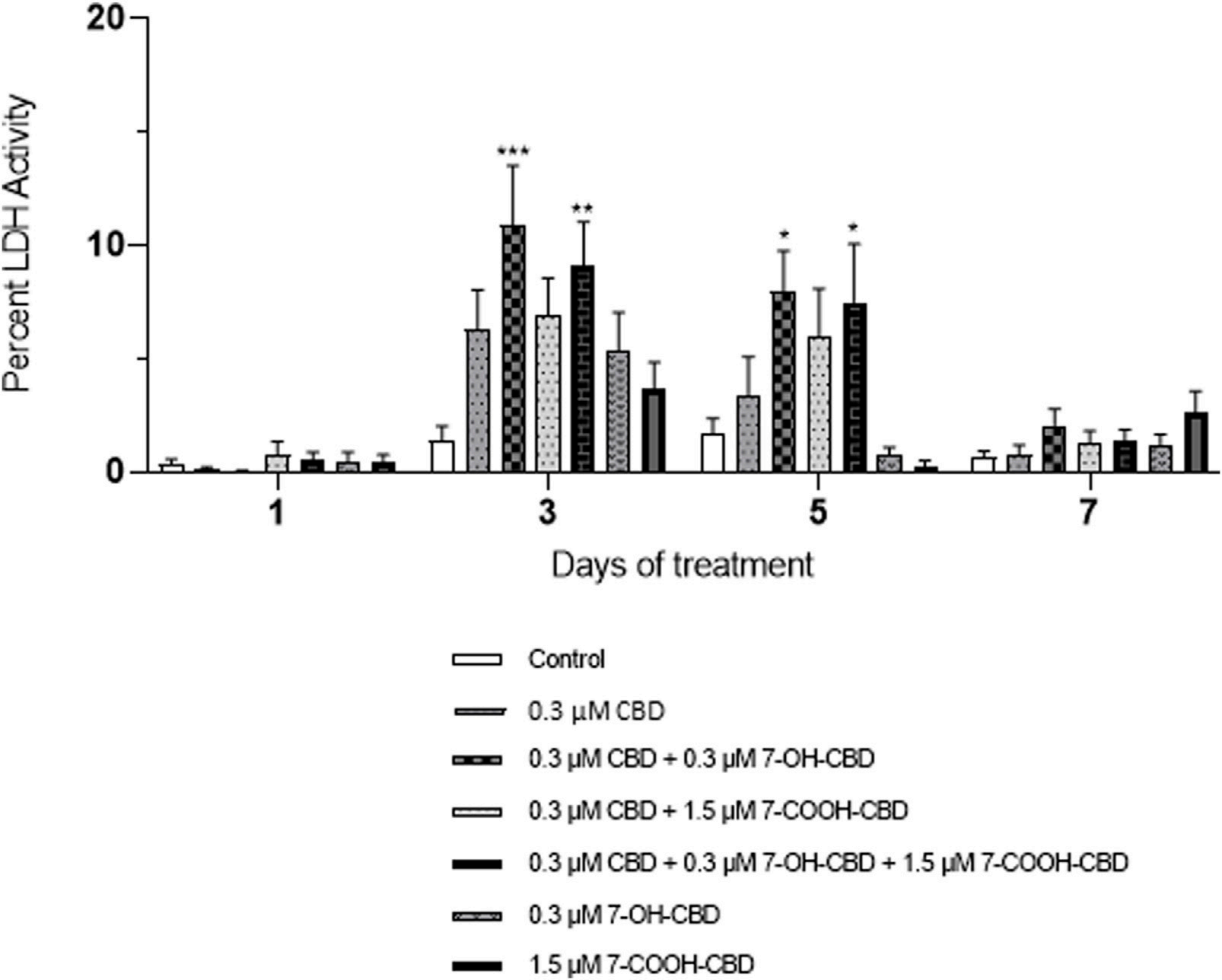
LDH assay of combination treatments. NSCs were exposed to 0.3 µM CBD and 7-OH-CBD, and 1.5 µM 7-COOH-CBD and their combinations. CBD and 7-OH-CBD additively increased LDH release starting from day 3. *P < 0.05; **P < 0.01; ***P < 0.001, compared with the control, n = 4–6. The experiment was repeated at least three times independently.

According to a pharmacokinetic study [18], CBD metabolites 7-OH-CBD and 7-COOH-CBD were detected soon after the CBD intake, and the terminal elimination half-life was 14–17 h for CBD, 14–19 h for 7-OH-CBD, and 25–30 h for 7-COOH-CBD after one dose of CBD, suggesting that the human brain could be exposed to CBD and its metabolites simultaneously after CBD intake. Therefore, in this study, NSCs were exposed to a combination of CBD, 7-OH-CBD and 7-COOH-CBD to assess whether CBD, 7-OH-CBD and 7-COOH-CBD had any additive or synergistic toxic effects on NSCs. NSCs were exposed to 0.3 µM CBD, 0.3 µM7-OH-CBD, and 1.5 µM 7-COOH-CBD individually or in combination. The concentrations of each compound were selected to be similar to the steady plasma concentrations of CBD, 7-OH-CBD and 7-COOH-CBD found in a clinical trial, in which subjects took 1,500 mg CBD twice a day for 6 days, with a single dose on the morning of day 7 [18]. Compared with the control group (0.1% DMSO), although the NSCs exposed to individual chemical had a higher level of LDH release after 3 days exposure, the elevation was not statistically significant. The LDH release was significantly higher in the group of NSCs exposed to a combination of CBD with 7-OH-CBD, with an increase of 9.6%. Exposure to CBD and the two metabolites stimulated LDH release from NSCs by 7.8% after 3 days exposure. There was a 6.3% increase of LDH release in the group treated with CBD and 7-OH-CBD, and a 5.8% increase after treatment of CBD and the two metabolites for 5 days (Figure 2), while the single drug did not make a significant difference (Figure 2).

### Drugs effects on NSC proliferation and cell cycle

**FIGURE 3:**
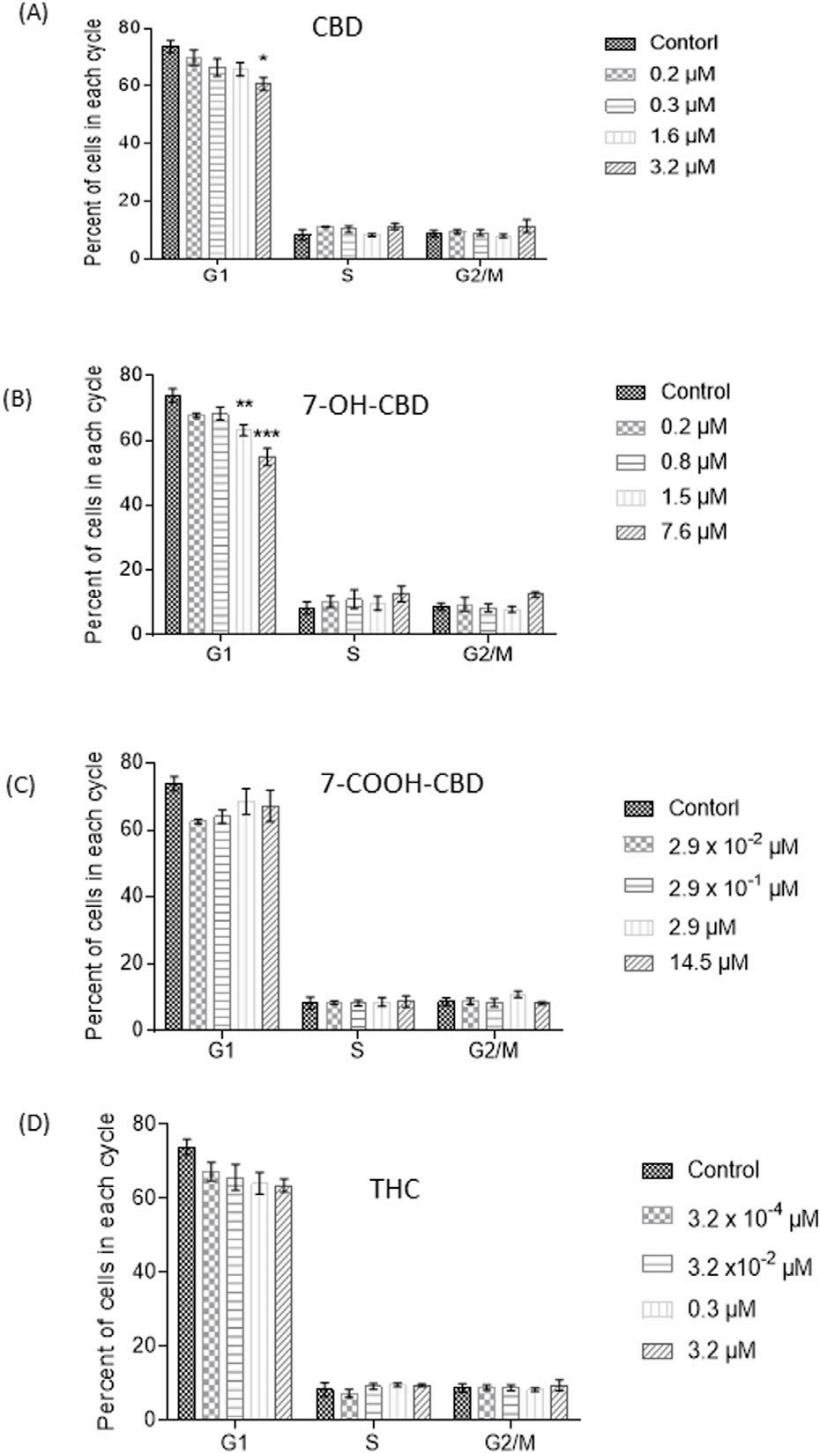
Cell cycle analysis after 24 h CBD, 7-OH-CBD, 7-COOH-CBD and THC exposures. Decreased diploid cell number in G1 phase was detected in 3.2 µM CBD **(A)**, 1.5 µM and 7.6 µM 7-OH-CBD **(B)** groups after 24 h exposure. The highest concentration of 14.5 µM 7-COOH-CBD **(C)** and 3.2 µM THC **(D)** did not cause a significant change of cell cycle. *P < 0.05; **P < 0.01; ***P < 0.001, compared with the control, n = 4–6. The experiment was repeated at least three times independently.

The cell cycle analysis did not observe any changes in S phase from any treated group, suggesting no obvious effect on NSC proliferation after 24 h exposure to the chemicals. The EdU assay showed similar results (data not shown). However, the number of G1 phase cells were reduced after exposure to 3.2 µM CBD by 13.1% (Figure 3A). Both 1.5 µM and 7.6 µM 7-OH-CBD caused reductions of G1 phase cells by 10.7% and 19.0%, respectively (Figure 3B), suggesting fewer diploid cells after exposure. THC of 3.2 µM also reduced G1 phase cell number (Figure 3D), but with a p value of 0.059. 7-COOH-CBD did not show a significant effect (Figure 3C).

### GSH levels in NSCs

**FIGURE 4:**
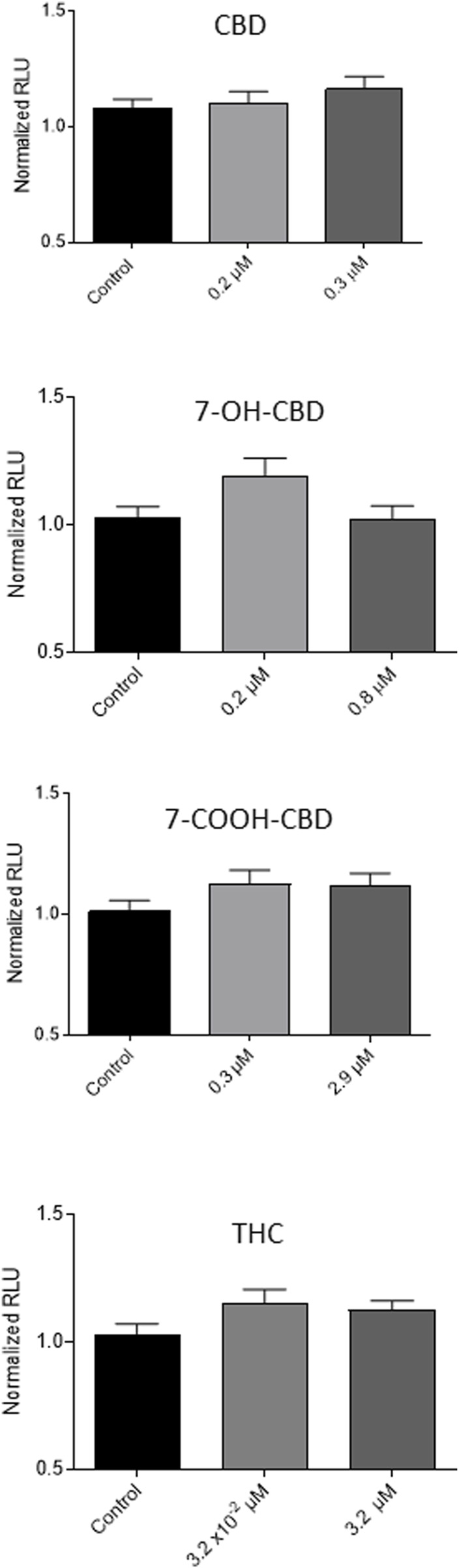
GSH levels after CBD, 7-OH-CBD, 7-COOH-CBD and THC exposures for 7 days. CBD at 0.2 and 0.3 µM, 7-OH-CBD at 0.2 and 0.8 µM, 7-COOH-CBD at 0.3 and 2.9 µM and THC at 3.2 × 10^−2^ and 3.2 µM did not make a significant change in the GSH levels in the exposed cells. N = 4–6. The experiment was repeated at least three times independently.

CBD has been reported to be an antioxidant [4, 36], while THC has been shown to be an antioxidant or to increase oxidative stress, depending on different conditions [37, 38]. Whether CBD and its main metabolites affect the redox status in NSCs was determined by the measurement of GSH levels in NSCs. After 7 days exposure, CBD at 0.2 and 0.3 µM, 7-OH-CBD at 0.2 and 0.8 µM, 7-COOH-CBD at 0.3 and 2.9 µM and THC at 3.2 × 10^−2^ and 3.2 µM did not alter GSH in NSCs significantly (Figure 4). The concentrations of each drug were selected based on the result of LDH assay, which did not show obvious cytotoxic effects on NSCs, except that 0.3 µM of CBD caused a small but significant increase of LDH release.

### NSC apoptosis detected by annexin V-PI staining and TUNEL assay

**FIGURE 5:**
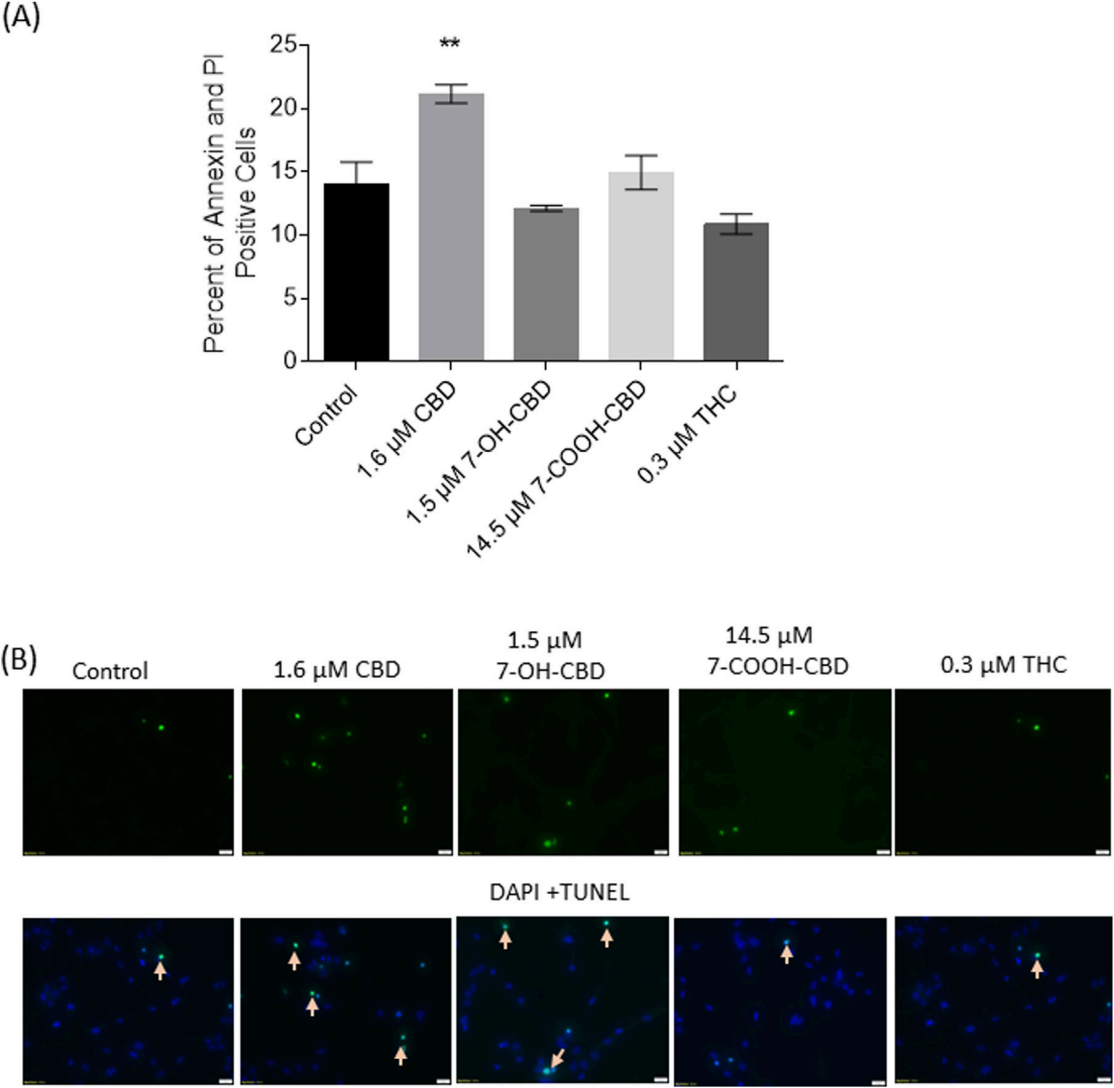
Cell death detected by Annexin V-PI staining and TUNEL assay. **(A)** Twenty-four-hour exposure to 1.6 µM CBD significantly elevated the number of both Annexin V^+^ and PI^+^ cells. **(B)** Images of TUNEL assay showed the drugs-induced apoptotic cells (indicated by arrows). **P < 0.01, compared with the control, n = 3. The experiment was repeated three times independently.

In this study, TUNEL assay and flow cytometry of Annexin V and PI staining were conducted to verify the toxic effects of cannabidiol and its main metabolites. The control group showed 14% of cells were Annexin V^+^ and PI^+^. There was a 7% increase of positive cells in 1.6 µM CBD group, suggesting CBD-induced cell death was mainly late-stage apoptosis after 24 h exposure. However, no significant increase of Annexin V^+^ and PI^+^ positive cells was detected in 1.5 µM 7-OH-CBD, 14.5 µM 7-COOH-CBD or 0.3 µM THC groups (Figure 5A). TUNEL positive cells were detected in each treated group. Although some dead cells detached during the experimental procedure, the CBD-treated group still showed obvious TUNEL positive cells. The other groups showed scattered TUNEL positive cells (Figure 5B).

### Expression levels of β-tubulin III, GFAP, OMG, caspase 3, CB1 and CB2 receptors after drug exposures

**FIGURE 6:**
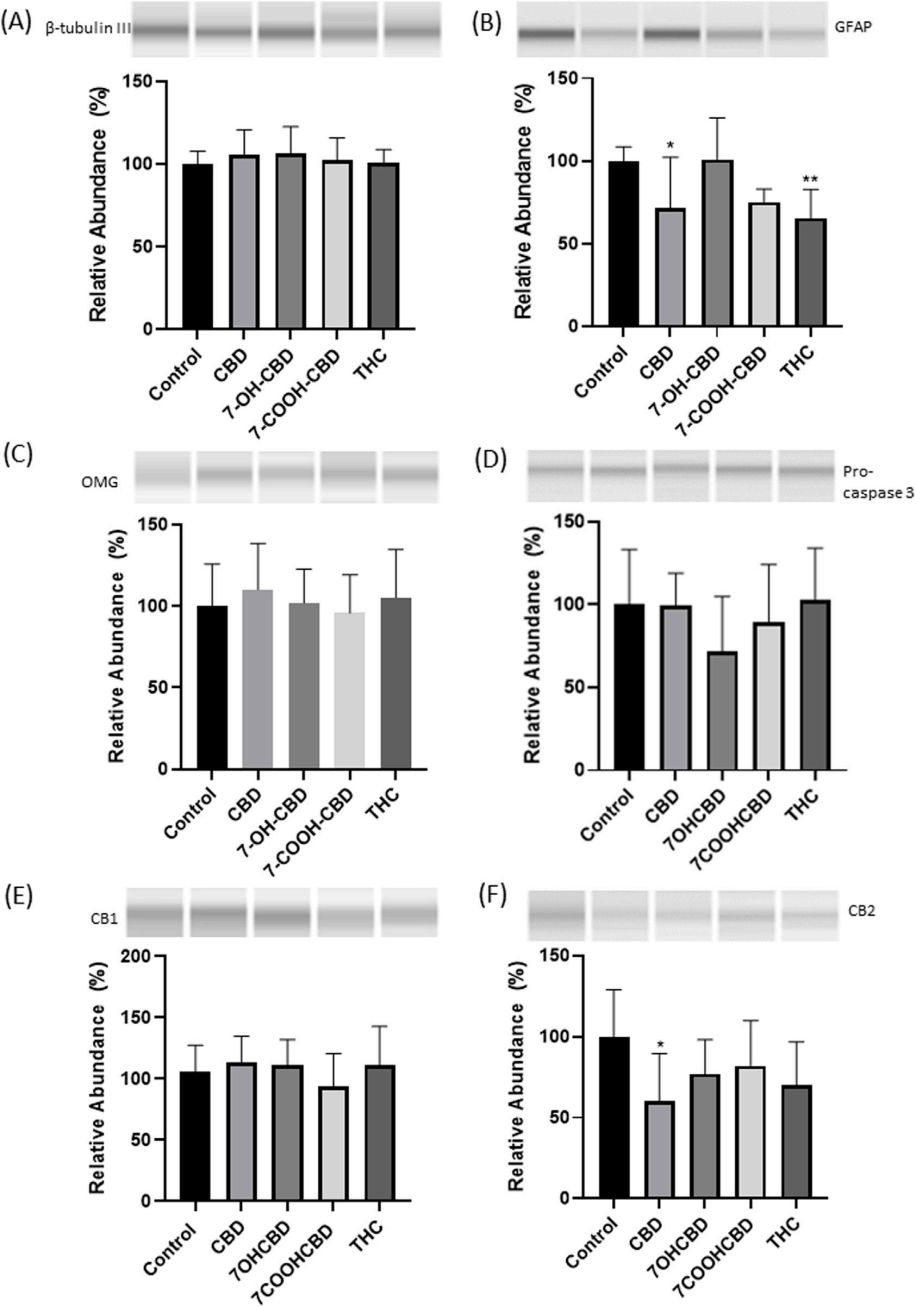
Western blots of β-tubulin III, GFAP, OMG, pro-caspase 3, CB1 and CB2 receptors in differentiated cells. No significant changes in the expression of β-tubulin III **(A)**, OMG **(C)**, pro-caspase 3 **(D)** or CB1 receptor **(E)**. GFAP expression was lower in CBD and THC exposed groups **(B)**. CB2 receptors expression was reduced in CBD exposed group **(F)**. *P < 0.05; **P < 0.01; n = 3–5. The experiment was repeated at least three times independently.

From the 4th day of differentiation, the cells were treated with 0.3 µM CBD, 0.2 µM 7-OH-CBD and 1.5 µM 7-COOH-CBD for 6 days, which were comparable to the steady concentrations detected in the human blood when the subjects took 1,500 mg CBD twice daily for 6 days [18]. It was reported that serum concentration of THC ranged from 13 to 63 ng/mL in cannabis smokers (from a 7% Δ9-THC content cigarette) 0–22 h post inhalation [39]. The range of individual peak concentrations of THC is 1.6–160 μg/L (1.6–160 ng/mL) [29]. Therefore, the differentiated cells were exposed to 0.3 µM THC. After 10 days differentiation, markers for neurons (β-tubulin III), astrocytes (GFAP) and oligodendrocytes (OMG) were detected by Western Blots (Figures 6A–C), suggesting NSCs have differentiated into neurons and glial cells. No active caspase 3 was detected. The expression of pro-caspase 3 was similar among groups (Figure 6D). Although the signals were not as strong as GFAP, β-tubulin III or OMG, CB1 (Figure 6E) and CB2 (Figure 6F) were detected, suggesting that differentiated cells expressed CB1 and CB2 receptors. CBD treatment resulted in decreased expression of GFAP and CB2 receptors on differentiated cells. THC treatment significantly reduced GFAP expression, while the reduction of CB2 receptors did not reach statistical significance. The CBD metabolites of 7-OH-CBD and 7-COOH-CBD did not cause significant changes in expression of β-tubulin III, GFAP, OMG, CB1 and CB2 after 6 days treatments.

## Discussion

CBD products are purported to treat numerous health conditions in the popular media, but in almost every instance lack approval from a regulatory agency. While there are studies reporting the beneficial effects of CBD [1, 2, 36, 40, 41], evidence on its adverse effects has also emerged [26, 27, 42]. Its interaction with other drugs is another concern [43]. When fetuses are exposed due to pregnant women consuming CBD, the fetal central nervous system (CNS) can be more vulnerable because of their incomplete development. There is a need to determine how much and how long CBD can be consumed before it may have any adverse effects on the developing human brain. Whalley et al. [44] observed interspecies variations in endocannabinoid signaling, implying possible species-specific inaccuracies if animal models are used to predict how CBD affects the human brain. Moreover, it is not possible to explore the effects of an early-life stressor such as CBD exposure in the human fetus. To obtain data from more relevant models, we purchased human NSCs that were collected from human fetal brain at gestational week 19 to conduct dose-response and time-course studies. Cultured NSCs can proliferate and differentiated *in vitro* [31, 32]. The present study detected strong expression of β-tubulin III, GFAP and OMG (Figures 6A-C), repeatedly confirming human NSC differentiation *in vitro*. Therefore, human NSCs can recapitulate some basic biological events happening in the developing human brain, allowing for the investigation of drug exposure events in a short period of time, and in a simplified system.

### Effects of CBD, its metabolites and THC on NSCs

A challenge for modeling the effects of CBD in animals is distinguishing the effects of CBD from those of its metabolites. The relative ratios of CBD metabolites in animal models (e.g., dogs, rats, etc.) are incomparable to those of humans [45–49]. Moreover, CBD concentrations in human blood vary depending on the doses, frequency and routes of administration, and the consumers’ healthy state, etc. Even diet change can influence CBD concentrations in blood [18]. There is a need to perform screening using different concentrations of CBD to estimate the consequence of brain exposure. In a clinical trial, Taylor et al. [18] measured CBD and CBD metabolites including 7-OH-CBD and 7-COOH-CBD concentrations in healthy volunteer blood after they took different doses of CBD (Epidiolex^®^). A dose of 1,500 mg/day CBD administration resulted in the plasma Cmax of CBD, 7-OH-CBD and 7-COOH-CBD of 292.4 ng/mL (0.9 μM), 238.7 ng/mL (0.7 μM) and 3,060 ng/mL (8.9 μM), respectively. When 4,500 mg/day of CBD was administered, the Cmax of CBD, 7-OH-CBD and 7-COOH-CBD in plasma reached 722.1 ng/ml, 404.8 ng/ml and 5,120 ng/ml, respectively [18]. There is no report on CBD or its metabolites concentrations in the human brain, but data from animal experiments showed CBD reached the brain with a relatively high concentration soon after it was orally administered [50]. Considering the lipophilic property of CBD, in the present study, we selected a series of concentrations for CBD and its metabolites to treat human NSCs, based on their concentrations detected in human blood and the animal brain. The utilization of NSCs helped to compare the relative toxicity of CBD, its main metabolites and THC, to predict their potential toxicity *in vivo*, which is difficult with whole animal studies. Our findings demonstrated that NSCs were vulnerable and sensitive to CBD and its metabolites as evidenced by increase cell death, after the exposure to various concentrations, at different time points. Of note, even lower concentrations of CBD and the metabolites showed slight yet significant cytotoxic effects after long term exposure (Figure 1), suggesting the potential risks of chronic exposure to CBD *in vivo*. THC concentrations in human blood vary dramatically when the amount and frequency of consumption are different [51, 52]. In this study, THC concentrations were chosen to include both low and high THC concentrations detected in humans [51, 52]. It seemed that THC had similar effects on NSC viability as those of CBD (Figure 1), although CBD does not have psychoactive effects. In addition to the effects of 7-OH-CBD in isolation, we observed additive effects of CBD and 7-OH-CBD on cell death (Figure 2), providing evidence that 7-OH-CBD has active effects on NSCs. It is well known that 7-COOH-CBD is the most abundant metabolite in plasma [18, 53]. Its direct effects on the human brain were not fully determined due to the different concentrations in human and animal models. We observed the cytotoxic effects of 7-COOH-CBD on NSCs (Figure 1), which has not been reported in other studies.

Oxidative stress is often associate with cell death [54, 55]. Cellular GSH levels are an indicator of redox status. It was reported that high levels of GSH were essential for stem cells [56]. CBD was reported to have antioxidative and neuroprotective effects mediated by various mechanisms [41, 57–59]; THC has demonstrated different effects on redox homeostasis in different situations [37, 38]. In this study, we measured cellular GSH levels to understand NSC redox status after exposure to CBD, its metabolites, and THC for 7 days. The cellular GSH levels were not dramatically changed (Figure 4). It seemed that these drugs at subtoxic concentrations (except 0.3 µM CBD causing a subtle but detectable change in LDH release) did not significantly affect the redox status in NSCs.

### Effects of CBD, its metabolites and THC on differentiated cells

In addition to NSCs, neurons and glial cells differentiated from NSCs are exposed to drugs and chemicals after they enter the fetal brain. In the present study, to determine responses of neurons, astrocytes, and oligodendrocytes after drug exposure, NSCs that had been differentiated for 3 days were treated with 0.3 µM CBD, 0.2 µM 7-OHCBD, 1.5 µM 7-COOHCBD, and 0.3 µM THC respectively for 6 days. No visible cell death was observed during the 6 days treatment, nor altered caspase 3 expression was detected in the differentiated cells, indicating these drugs did not cause significant death of developing neurons and glial cells. Western-blot results did not identify changes in β-tubulin III expression (Figure 6A), suggesting neuronal differentiation was not significantly affected by CBD, its metabolites or THC at the exposed concentrations. However, GFAP expression was decreased in CBD and THC-treated groups (Figure 6B). There is research work demonstrating that THC changed GFAP expression in the animal brain during development [60–63]. A recent study by Landucci et al. presented that CBD reduced GFAP expression in CA1 region of the developing rat hippocampus [61]. Not only in early development, GFAP expression was adjusted in adolescence or adulthood after THC exposure [64]. In the present study, since no significant cell death was observed, reduced GFAP expression could suggest that the cytoskeletal structure of astrocytes was modified, which could affect astrocyte maturation and functions. Our observations and those from others indicated that the GFAP expression seems to be quite sensitive and indicative of CBD and THC exposure. In contrast to their adverse effects on NSCs, CBD metabolites of 7-OH-CBD and 7-COOH-CBD did not significantly reduce GFAP expression, although 7-COOH-CBD showed the tendency (Figure 6B).

Both CB1 and CB2 receptors have been detected on neurons and astrocytes; and CB2 receptors are found to be present on microglia too [65–69]. It has been demonstrated that CB1 and CB2 receptors are expressed in the developing brain [70]. In the present study, with the limitation of available antibodies against CB1 and CB2 receptors, it was difficult to locate CB1 and CB2 receptors on neurons or astrocytes using immunocytochemical staining, although Western blots detected CB1 and CB2 receptors expression on differentiated cells. The relative weak expression of CB2 receptor of the Western blots suggested the low abundance of CB2 receptor expression during early brain development. CBD induced down-regulation of GFAP and CB2 receptors. Therefore, it was hypothesized that CBD could have interacted with astrocytes during astrocyte differentiation, causing modulation of astrocyte functions and CB2 receptor expression on astrocytes. While the consequences of the reduced CB2 receptor expression in the developing brain have yet to be elucidated, it was reported that decreased CB2 receptor expression could increase seizure susceptibility and cause a deficiency of social memory in mice [71, 72]. It will be an intriguing topic to understand how cannabinoids would affect CB2 receptors during the brain development, and whether such effects may alter brain functions in adulthood.

### Summary

In this study, we performed an evaluation of key cannabinoids on the effects of NSC biology. Our data has demonstrated the adverse effects of CBD, its metabolites, and THC on NSCs and differentiated cells, indicating their toxic effects on the human brain at an early developmental stage. Observed effects of 7-OH-CBD and 7-COOH-CBD on NSCs highlighted their possible bioactivity *in vivo*. The cell cycle assay provided additional evidence that these drugs reduced the number of diploid cells, indicating cell death. We focused on some primary endpoints after the differentiated cells were exposed to drugs for 6 days, and detected changes in GFAP and CB2 receptors. Although more areas need to be explored, the present findings have provided evidence that CBD and its main metabolites at concentrations comparable to those detected in human blood may have adverse effects on the developing brain *in vivo*, especially after long-term exposure. Moreover, the comparative analysis of CBD and its key metabolites will also help to put findings from non-clinical studies, where metabolite profiles may not match that observed in humans, into context.

## Data Availability

The original contributions presented in the study are included in the article/supplementary material, further inquiries can be directed to the corresponding author.
